# Effects of dietary tributyrin on intestinal mucosa development, mitochondrial function and AMPK-mTOR pathway in weaned pigs

**DOI:** 10.1186/s40104-019-0394-x

**Published:** 2019-11-25

**Authors:** Chunchun Wang, Shuting Cao, Zhuojun Shen, Qihua Hong, Jie Feng, Yan Peng, Caihong Hu

**Affiliations:** 1 Key Laboratory of Molecular Animal Nutrition, Ministry of Education, Animal Science College, Zhejiang University, Yu Hang Tang Rd No. 866, Hangzhou, 310058 People’s Republic of China; 2 Shanghai Menon Animal Nutrition Technology Co. Ltd., Shanghai, 201807 China

**Keywords:** AMPK-mTOR signaling pathway, Intestinal mucosa development, Mitochondrial function, Tributyrin, Weaned pigs

## Abstract

**Background:**

The objective of this experiment was to investigate the influence of dietary tributyrin on intestinal mucosa development, oxidative stress, mitochondrial function and AMPK-mTOR signaling pathway.

**Methods:**

Seventy-two pigs were divided into two treatments and received either a basal diet or the same diet supplemented with 750 mg/kg tributyrin. Each treatment has six replicates of six pigs. After 14 days, 6 pigs from each treatment were selected and the jejunal samples were collected.

**Results:**

Results showed that supplemental tributyrin increased (*P* < 0.05) villus height and villus height: crypt depth of weaned pigs. Pigs fed tributyrin had greater (*P* < 0.05) RNA/DNA and protein/DNA ratios than pigs on the control group. The mRNA levels of sodium glucose transport protein-1 and glucose transporter-2 in the jejunum were upregulated (*P* < 0.05) in pigs fed the tributyrin diet. Dietary tributyrin supplementation lowered (*P* < 0.05) the malondialdehyde and hydrogen peroxide (H_2_O_2)_ content in jejunum, enhanced (*P* < 0.05) the mitochondrial function, as demonstrated by decreased (*P* < 0.05) reactive oxygen species level and increased (*P* < 0.05) mitochondrial membrane potential. Furthermore, tributyrin increased (*P* < 0.05) mitochondrial DNA content and the mRNA abundance of genes related to mitochondrial functions, including peroxisomal proliferator-activated receptor-γ coactivator-1α, mitochondrial transcription factor A, nuclear respiratory factor-1 in the jejunum. Supplementation with tributyrin elevated (*P* < 0.05) the phosphorylation level of AMPK and inhibited (*P* < 0.05) the phosphorylation level of mTOR in jejunum compared with the control group.

**Conclusions:**

These findings suggest that dietary supplementation with tributyrin promotes intestinal mucosa growth, extenuates oxidative stress, improves mitochondrial function and modulates the AMPK-mTOR signal pathway of weaned pigs.

## Background

Piglets are often subjected to nutritional, physiological and immunological stresses during the weaning process [1]. It has been determined that weaning can quickly lead to the intestinal-mucosal injury, such as villus atrophy and crypt hyperplasia and further impair intestinal absorption function [2–5]. Butyrate, as a SCFA (short chain fatty acid), has gained much attention due to its rewarding effects on cellular energy metabolism and intestinal homeostasis [6]. Butyrate is rapidly absorbed across the luminal membranes of intestinal epithelial cells under the action of butyrate transporters [7]. Substantial evidence has reported that butyrate plays a potential role in affecting epithelial cell growth and differentiation, and repairing intestinal injury [8–10]. Tributyrin, containing three molecules of butyrate, has also been reported to maintain intestinal mucosa normal function [11, 12]. Therefore, it is necessary to study the beneficial effects of butyrate or tributyrin on intestinal development and absorption function in weaned piglets.

The intestine requires a lot of energy to renew and repair the damaged intestinal mucosa [13], and mitochondria are responsible for the main source of cellular energy [14]. Mitochondria are the primary site of reactive oxygen species (ROS) generation, and at the same time, they are highly susceptible to ROS [15]. Our previous study has found that weaning caused oxidative stress and severely impaired mitochondrial function [16]. Recently, Xing et al. [17] reported that in HepG2 cells, sodium butyrate alleviated oxidative injury and enhanced mitochondrial function. Besides, several papers reported that butyrate was effective in relieving intestinal oxidative stress [18, 19]. Nevertheless, no information is available about the effects of butyrate or tributyrin on oxidative stress and mitochondrial function of intestine in weaned pigs.

AMPK (AMP-activated protein kinase), a sensor of energy state, is responsible for regulating mitochondrial function and cell energy metabolism [20]. Activation of AMPK results in the inhibition of mTOR (mammalian target of rapamycin), a downstream regulator of AMPK that senses energy and stress [21]. Substantial evidence has suggested that the AMPK-mTOR pathway acted as an important role in modulating oxidative stress [22–24]. However, the effect of butyrate or tributyrin on AMPK-mTOR pathway has not been reported so far.

Accordingly, we hypothesized that dietary tributyrin could enhance intestinal mucosa development, improve mitochondrial function and influence AMPK-mTOR signaling pathway of weaned pigs. Our objective was aimed at exploring the effect of dietary tributyrin on intestinal mucosa growth, mitochondrial function as well as AMPK-mTOR signaling pathway of weaned pigs.

## Methods

### Experimental design

Seventy-two pigs (Duroc × Landrace × Yorkshire, body weight (BW) of 6.8 kg, weaned at 24 ± 1 days of age), were divided into two treatments according to their initial BW and sex. Each treatment has six replicates of six pigs (3 barrows and 3 gilts). Two treatments received basal diet or the same diet supplemented with 750 mg/kg tributyrin (provided by Shanghai Menon Feed Co. Ltd., Shanghai, China). The formula for the diets is in compliance with NRC (2012) (Table 1). Pigs had free to drink and feed.

**Table 1:** Ingredient and composition of diets on an as-fed basis

Item	
Ingredients, g/kg	
Corn	455
Wheat middling	80
Soybean meal	170
Fish meal	30
Spray-dried plasma protein	30
Dried whey	150
Soybean oil	15
Dicalcium phosphate	6
Limestone	5
Sodium chloride	1
*L*-Lysine HCl	5
*DL*-Methionine	1.2
*L*-threonine	1.7
Sucrose	30
Vitamin-mineral premix^a^	20.1
Analyzed composition, g/kg	
Digestible energy^b^, MJ/kg	14.9
Crude protein	203.4
Lysine	14.3
Methionine	4.7
Calcium	6.9
Total phosphorus	6.2

^a^Provided the following per kilogram of diet: vitamin A, 8750 IU; vitamin D_3_, 2500 IU; vitamin E, 25 IU; vitamin K_3_, 2.5 mg; vitamin B_1_, 2.5 mg; vitamin B_2_, 6.25 mg; vitamin B_6_, 2.5 mg; vitamin B_12_, 25 μg; *D*-Biotin, 100 μg; folic acid, 1.25 mg; nicotinamide, 25 mg; *D*-pantothenic acid, 12.5 mg; Zn, 80 mg; Fe, 80 mg; Cu, 20 mg; Mn, 40 mg; I, 0.15 mg; Se, 0.3 mg; Co, 0.3 mg

^b^Digestible energy was calculated from data provide by Feed Database in China (2012)

### Sample collection

After 14 days, 6 pigs (3 barrows and 3 gilts) from each treatment (1 pig per pen) were euthanized with an ear intravenous injection of sodium pentobarbital (200 mg/kg BW) (Sigma-Aldrich, St. Louis, MO) and the gastrointestinal tract was rapidly removed. Proximal jejunal samples were fixed in 4% paraformaldehyde for measurement of intestinal mucosa architecture. Segments of the proximal jejunum were immediately gained to isolate intestinal mitochondria. The mucosa from the adjacent jejunum were harvested and placed in liquid nitrogen for further analysis.

### Measurement of intestinal mucosa architecture

Segments for morphological study were fixed in 4% paraformaldehyde and then embedded in paraffin wax. Sections of 5 μm were cut and stained with hematoxylin and eosin. Crypt depth and villus height were measured in three intestinal cross sections with at least 10 well-oriented crypt villus units for each cross-section using an image processing and analysis system (Leica Imaging Systems, Cambridge, UK) and averaged for each sample.

### Measurement of mucosal DNA, RNA and protein

The jejunal DNA, RNA and protein were collected from snap-frozen mucosal samples, with TRI Reagent-RNA/DNA/Protein isolation reagent (TaKaRa Biotechnology, Dalian, China). The concentrations were measured according to Jiao et al. [25].

### Determination of antioxidative enzyme activities

Antioxidant parameters, including superoxide dismutase (SOD), catalase (CAT), malondialdehyde (MDA) and hydrogen peroxide (H_2_O_2_) in jejunum were determined with commercially available kits (Nanjing Jiancheng Bioengineering Institute, Nanjing, China).

### Isolation of mitochondria

Fresh jejunal mucosa was prepared to isolate mitochondria. The process was in compliance with the rules of mitochondria isolation kit (Beyotime Institute of Biotechnology). After intestinal mucosa was homogenized in MSH buffer (10 mmol/L HEPES, pH 7.5, containing 200 mmol/L mannitol, 70 mmol/L sucrose, 1.0 mmol/L egtazic acid and 2.0 mg/mL serum albumin), the homogenate was centrifuged at 1000×*g* for 10 min at 4 °C. And then the collected supernatant was centrifuged at 3500×*g* for 10 min at 4 °C to acquire mitochondrial pellet [26].

### Mitochondrial reactive oxygen species (ROS) content

The fluorescent probes (2′,7′-dichlorohydro-fluorescein diacetate, DCFH-DA) were used for detecting mitochondria ROS content as previously described [27]. DCFH-DA can pass through the cell membrane and be hydrolyzed into DCFH. ROS can oxidize non-fluorescent DCFH into DCF, which has a maximum absorption peak at an excitation wavelength of 485 nm and an emission wavelength of 525 nm, and the intensity is proportional to the level of ROS. The fluorescence of DCF was assayed using a fluorescence microplate reader (FLx800, Bio-Tek Instruments, Inc.). The results were indicated as fold changes of the control.

### Mitochondrial membrane potential (∆Ψm)

The changes of ∆Ψm were assayed by ∆Ψm assay kit with JC-1 (Beyotime Institute of Biotechnology). The isolated mitochondria were strained with JC-1 at 37 °C for 15 min, and then the fluorescence intensity was assayed using a fluorescence microplate reader (FLx800, Bio-Tek Instruments, Inc.). JC-1 monomer form (green) fluorescence was excited at 485 nm, and the emission was detected at 530 nm. JC-1 aggregate form (red) fluorescence was excited at 485 nm, and emission fluorescence was detected at 590 nm. The ratio of red and green fluorescence values was used to reflect the ∆Ψm [16].

### Determination of mitochondrial DNA (mtDNA) content

The mtDNA numbers relative to genomic DNA were assayed with co-amplification of the mitochondrial DNA-loop (*mt D-loop*) and the nuclear-encoded *β-actin* gene using RT- PCR according to the previous steps [28]. Total DNA was extracted from proximal jejunal mucosa using a TIANamp Stool DNA Kit (Tiangen Biotech, Beijing, China). For the *mt D-loop* the primers were forward 5′-GATCGTACATAGCACATATCATGTC-3′, reverse 5′-GGTCCTGAAGTAAGAACCAGATG-3′. For *β-actin* the primers were forward 5′-CCCCTCCTCTCTTGCCTCTC-3′, reverse 5′- AAAAGTCCTAGGAAAATGGCAGAAG-3.

### mRNA expressions of nutrient transporter genes and mitochondrial function-related genes

Total RNA of jejunal mucosa was obtained with the Trizol reagent (TaKaTa, Dalian, China) according to the kit’s direction. RNA concentration and purity were assayed by a Nano Drop spectrophotometer (ND-2000; NanoDrop Technologies, Wilmington, DE). Reverse transcription was performed with the PrimeScripte RT reagent kit (TaKaRa Biotechnology, Dalian, China). Quantitative RT-PCR was performed on a StepOne Plus real-time PCR system (Applied Biosystems, Foster City, CA) using SYBR Green Master mix (Promega, Madison, WI) as described by Liu et al. [29]. The genes include sodium glucose transport protein-1 (*SGLT1*), glucose transporter-2 (*GLUT2*), Na^+^-dependent neutral amino acid transporter 2 (*ASCT2*), y^+^ L-type amino acid transporter 1 (*y*^*+*^ *LAT1*), dipeptide transporter 1 (*PepT1*), peroxisomal proliferator-activated receptor-γ coactivator-1α (*PGC-1α*), mitochondrial transcription factor A (*TFAM*) and nuclear respiratory factor-1(*NRF-1*). Primers are exhibited in Table 2. The β-actin was used as a reference gene to normalize mRNA levels of each target gene. The 2^−ΔΔCt^ method was used to analyze the relative expression, calculated relative to the values from the control group.

**Table 2:** Primers used for real-time quantitative PCR

Primer name	Primer sequence	Size, bp	Accession numbers
*SGLT1*	F:5′-TCATCATCGTCCTGGTCGTCTC-3′ R:5′-CTTCTGGGGCTTCTTGAATGTC-3′	144	M34044.1
*GLUT2*	F:5′-ATTGTCACAGGCATTCTTGTTAGTCA-3′ R:5′-TTCACTTGATGCTTCTTCCCTTTC-3′	273	NM_001097417
*ASCT2*	F:5′-CTGGTCTCCTGGATCATGTGG-3′ R:5′-CAGGAAGCGGTAGGGGTTTT-3′	172	DQ231578.1
*y*^+^*LAT1*	F:5′-TTCTCTTACTCGGGCTGGGA-3′ R:5′-GCGCCATGAGACCATTGAAC-3′	400	EU047705.1
*PepT1*	F:5′-CAGACTTCGACCACAACGGA-3′ R:5′-TTATCCCGCCAGTACCCAGA-3′	99	NM_214347.1
*PGC-1α*	F:5′-CCCGAAACAGTAGCAGAGACAAG-3′ R:5′-CTGGGGTCAGAGGAAGAGATAAAG-3′	111	NM_213963.2
*TFAM*	F:5′-GGTCCATCACAGGTAAAGCTGAA-3′ R:5′-ATAAGATCGTTTCGCCCAACTTC-3′	167	AY923074.1
*NRF-1*	F:5′-GCCAGTGAGATGAAGAGAAACG-3′ R:5′-CTACAGCAGGGACCAAAGTTCAC-3′	166	AK237171.1
*β-actin*	F:5′-GGATGCAGAAGGAGATCACG-3′ R:5′-ATCTGCTGGAAGGTGGACAG-3′	130	DQ845171.1

SGLT1: sodium-glucose transporter 1; GLUT2: glucose transporter type 2; ASCT2: Na^+^-dependent neutral amino acid transporter 2; y^+^ LAT1: y^+^ L-type amino acid transporter 1; PepT1: dipeptide transporter 1; PGC-1α: peroxisomal proliferator-activated receptor-γ coactivator-1α; TFAM: mitochondrial transcription factor A; NRF-1: nuclear respiratory factor-1

### Western blot assay

The steps are in accordance with the procedures of Hu et al. [5]. Sample in the jejunal mucosa was performed with SDS-PAGE and transferred to PVDF membranes. The membrane was blocked for 120 min at 25 °C, and then incubated with primary antibodies overnight at 4 °C. The membrane was rinsed and then incubated with the secondary antibodies for 60 min at 25 °C. The antibodies, including p-AMPK, AMPK, p-mTOR, mTOR, GAPDH and HRP-conjugated anti-rabbit Ab were purchased in Santa Cruz Technology Inc. (Santa Cruz, CA). The signal was displayed with Chemi Scope 3400 (ClinxScience Instruments, Shanghai, China) and the protein band value was counted with Image J analysis software.

### Statistical analysis

Data were analyzed by Student’s *t*-test with SPSS 22.0 statistical package (SPSS Inc., Chicago, IL). The significance value and a trend toward difference were set at levels of *P* < 0.05 and *P* < 0.10, respectively.

## Results

### Effect of tributyrin on jejunal mucosa architecture of weaned pigs

Intestinal mucosa architecture is exhibited in Table 3. The weaning pigs fed with tributyrin showed higher (*P* < 0.05) jejunal villus height and villus height: crypt depth in comparison to the control. The crypt depth had no difference in two treatments (*P* > 0.05).

**Table 3:** Effects of tributyrin on jejunal mucosa architecture of weaned pigs

Item	Control	Tributyrin	SEM	*P*
Villus height, μm	384.29	444.53	8.37	0.001
Crypt depth, μm	245.88	222.34	9.57	0.113
Villus height: Crypt depth	1.58	2.02	0.09	0.007

Values are mean and pooled SEM, *n* = 6

Differences were considered significant at *P* < 0.05

### Effect of tributyrin on jejunal DNA, RNA and protein of weaned pigs

Data about the concentrations of DNA, RNA and protein of weaned pigs are summarized in Table 4. Tributyrin increased (*P* < 0.05) jejunal RNA/DNA ratio as well as protein/DNA ratio in weaned pigs. No alteration was observed in the DNA concentrations between the two groups.

**Table 4:** Effects of tributyrin on DNA, RNA and protein concentrations in the jejunum of weaned pigs

Item	Control	Tributyrin	SEM	*P*
DNA, mg/g	0.61	0.67	0.05	0.373
RNA/DNA, g/g	5.54	6.97	0.33	0.012
Protein/DNA, g/g	94.57	117.55	4.40	0.004

Values are mean and pooled SEM, *n* = 6

Differences were considered significant at *P* < 0.05

### Effect of tributyrin on jejunal mRNA expression of nutrient transporter of weaned pigs

As shown in Table 5, dietary tributyrin induced an increase (*P* < 0.05) in *SGLT1* and *GLUT2* mRNA level, and had a trend (*P* < 0.1) to raise mRNA level of *ASCT2* in the jejunal mucosa of weaned pigs.

**Table 5:** Effects of tributyrin on mRNA levels of nutrient transporter genes in jejunum of weaned pigs

Item ^a^	Control	Tributyrin	SEM	*P*
*SGLT1*	1.00	1.97	0.16	0.002
*GLUT2*	1.00	1.68	0.17	0.017
*ASCT2*	1.00	1.52	0.18	0.075
*y*^+^*LAT1*	1.00	1.27	0.16	0.275
*PepT1*	1.00	1.31	0.15	0.183

^a^SGLT1: sodium-glucose transporter 1; GLUT2: glucose transporter type 2; ASCT2: Na^+^-dependent neutral amino acid transporter 2; y^+^ LAT1: y^+^ L-type amino acid transporter 1; PepT1: dipeptide transporter 1

Values are mean and pooled SEM, *n* = 6

Differences were considered significant at *P* < 0.05

### Effects of tributyrin on jejunal antioxidant indicators of weaned pigs

Table 6 indicates that the contents of MDA and H_2_O_2_ in jejunum were reduced (*P* < 0.05) in pigs fed tributyrin compared to those in control. However, no significant alteration (*P* > 0.05) was observed in activities of SOD and CAT of jejunum between the two groups.

**Table 6:** Effects of tributyrin on antioxidant indicators in the jejunum of weaned pigs

Item^a^	Control	Tributyrin	SEM	*P*
SOD, U/mg protein	58.21	67.36	3.28	0.076
CAT, U/mg protein	8.72	10.17	0.79	0.221
MDA, nmol/g protein	0.65	0.31	0.08	0.014
H_2_O_2_, mmol/g protein	17.38	12.48	1.27	0.021

^a^ *SOD* superoxide dismutase, *CAT* catalase; *MDA* malondialdehyde; *H*_*2*_*O*_*2*_ hydrogen peroxide

Values are mean and pooled SEM, *n* = 6

Differences were considered significant at *P* < 0.05

### Effect of tributyrin on jejunal mitochondrial ROS and △Ψ of weaned pigs

**Fig. 1:**
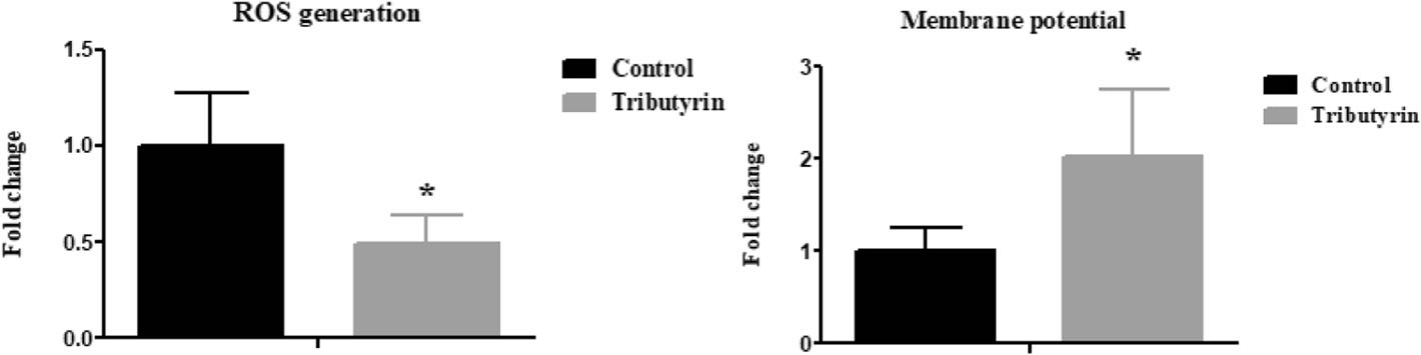
Effects of tributyrin on mitochondrial ROS production and ∆Ψm in jejunum of weaned pigs. Values are means and SD represented by vertical bars. *Differences were considered significant at *P* < 0.05. The ROS production and ∆Ψm were expressed as fold changes, calculated relative to the control group. ROS: reactive oxygen species; ∆Ψm: mitochondrial membrane potential

Figure 1 shows that in comparison with the control, tributyrin significantly reduced (*P* < 0.05) mitochondrial ROS level and raised (*P* < 0.05) △Ψ.

### Effect of tributyrin on jejunal mRNA expression of mitochondrial function-related genes of weaned pigs

**Fig. 2:**
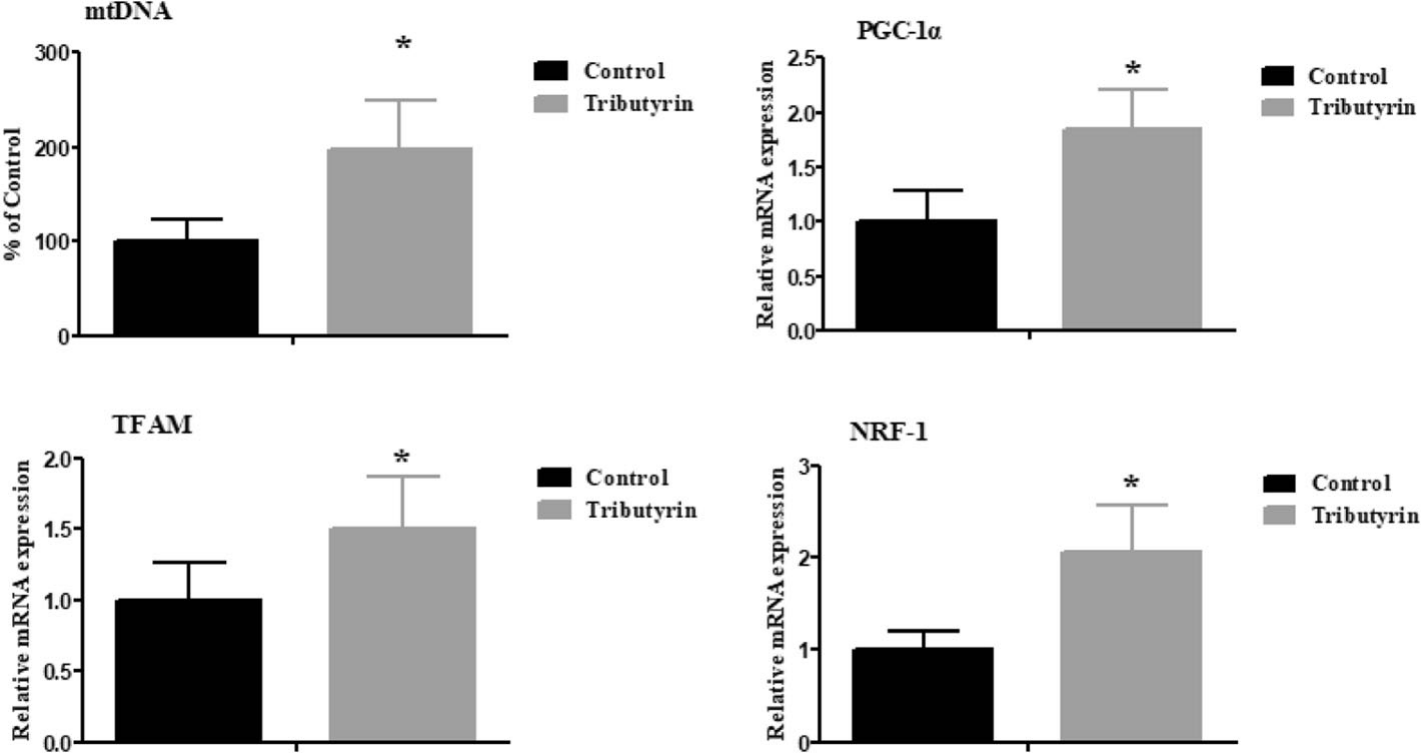
Effects of tributyrin on mtDNA and mRNA levels of mitochondrial function-related genes of weaned pigs. Values are means and SD represented by vertical bars. *Differences were considered significant at *P* < 0.05. mtDNA: mitochondrial DNA; PGC-1α: peroxisomal proliferator-activated receptor-γ coactivator-1α; TFAM: mitochondrial transcription factor A; NRF-1: nuclear respiratory factor-1

Figure 2 shows that tributyrin increased (*P* < 0.05) mtDNA copy numbers as well as mRNA levels of mitochondrial function-related genes, including *PGC-1α*, *TFAM* and *NRF-1*.

### Effect of tributyrin on AMPK-mTOR signaling pathway in the jejunum of weaned piglets

**Fig. 3:**
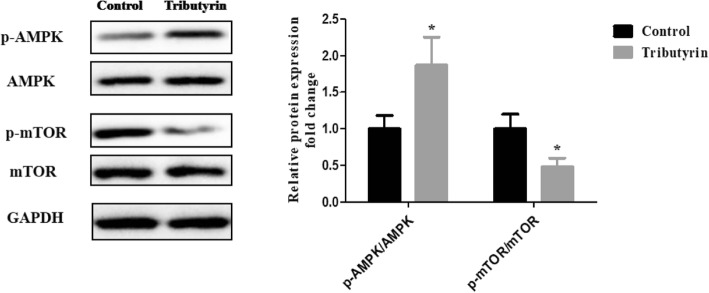
Effects of tributyrin on AMPK- mTOR signal pathway in jejunum of weaned pigs. The bands are representative blots from one of six pigs. The values are calculated as the ratios of their phosphorylation levels (p-AMPK, p-mTOR) and the total levels. Values are means and SD represented by vertical bars. *Differences were considered significant at *P* < 0.05

Figure 3 shows the effect of tributyrin on the AMPK-mTOR signaling pathway. Compared to the control, supplemental tributyrin effectively elevated (*P* < 0.05) the ratio of the phosphorylated AMPK to total AMPK (p-AMPK/AMPK), while decreased (*P* < 0.05) the ratio of the phosphorylated mTOR to total mTOR (p-mTOR/mTOR).

## Discussion

Weaning pigs are often confronted with changes of intestinal architecture, including villus atrophy and crypt hyperplasia [5]. Butyrate, a volatile fatty acid, plays a significant part in enhancing intestinal mucosa architecture and promoting intestinal growth [30]. This current study demonstrated that tributyrin increased villus height as well as villus height: crypt depth, suggesting that weaning-induced injury to jejunal mucosa architecture was alleviated by addition with tributyrin. Similar to our results, Dong et al. [11] showed that in the intrauterine growth restriction pigs, dietary tributyrin increased villus height: crypt depth of the duodenum and jejunum, and lowed crypt depth of duodenum. The protein level, the ratio of RNA to DNA and protein to DNA in the intestinal mucosa are valuable biological parameters to assess intestinal growth and development [25, 31]. DNA concentration represents the mitosis rate to renew columnar epithelial cell, RNA/DNA and protein/DNA signifies cell efficiency and protein synthesis efficiency, respectively [25]. In this research, dietary tributyrin elevated the jejunal RNA/DNA and protein/DNA, which is beneficial for the small intestinal growth of weaned pigs. Similarly, another study found that SCFAs were trophic to the intestinal mucosa as evidenced by the increased DNA, RNA and protein concentration in intestinal mucosa of rats [32]. Monocarboxylate transporter 1 (MCT-1) plays an important role in the transport of monocarboxylates across cell membrane [33]. It has been identified in the small intestine [34] and colon [35]. An earlier research reported that in cultured colonic epithelial cells, treatment with sodium butyrate caused a concentration- and time-dependent upregulation of MCT1 mRNA and protein [36]. These results indicated that dietary tributyrin may enhance the expression of butyrate transporters in the jejunum, thereby promoting the intestinal mucosa development.

Weaned piglets need a huge amount of nutrients to meet the rapid renewal and growth of the intestine. The absorption of nutrients mainly depends on specific transporters that transport nutrients across the intestinal epithelium [4]. However, few researches studied the action of tributyrin in intestine absorption function of weaned pigs. Therefore, we observed the effects of tributyrin on mRNA expressions of intestinal nutrient transporters (*SGLT1*, *GLUT2*, *ASCT2*, *y*^+^*LAT1*, *PepT1*). *SGLT1* is a rate-limiting step to transport glucose into the absorptive enterocyte, and *GLUT2* transported glucose from intestinal cell into the blood circulation [37]. *ASCT2* and *LAT1*, in charge for transporting neutral amino acid, have a principal part in the growth and proliferation of intestinal cell [38]. *PepT1*, widely existed in intestinal epithelial cell, is responsible to transport dipeptide from the lumen into enterocyte [39]. In present study, we found that the jejunal *SGLT1* and *GLUT2* mRNA abundances were elevated by tributyrin. The result was analogous with an *in vitro* study using Caco-2, which found that butyrate treatment elevated the mRNA level of *GLUT2* [40]. Weaning piglets were confronted with innutrition and intestinal disorders due to the immature digestion and absorption systems [1], which might be part of the reason why tributyrin can promote the mRNA expressions of the nutrient transporters of weaning piglets. Accordingly, it is likely that tributyrin enhanced absorption function, thus transporting more nutrition to improve the intestinal mucosa architecture.

Several studies reported that tributyrin reduced intestinal oxidative stress in the ethanol-challenged colitis [41] and colon of dextran sodium sulphate-challenged mice [42]. However, there was little evidence about the antioxidant effect of tributyrin in pigs. Therefore, it is necessary to assess the effect of tributyrin on redox state in intestine of weaned pigs. Generally, CAT and SOD are regarded as two main antioxidant enzymes and have essential roles in the prevention of oxidative injury. Surprisingly, we found that dietary tributyrin had no significant effects on enzyme activities of CAT and SOD. However, Leonel et al. [42] found that tributyrin supplementation lowered the H_2_O_2_ level, increased the SOD and CAT activities in dextran sodium sulphate-induced colitis of mice. Ma et al. [18] reported that after treatment with sodium butyrate, the levels of SOD, GSH-Px (glutathione peroxidase) and GSH (glutathione) increased while MDA level decreased in scratched IPEC-J2 cell. This discrepancy could be explained by the diverse trial conditions, animal species and animal model. MDA concentration in tissues and serum has been considered as biomarkers of oxidative injury [43]. In our study, the results showed that dietary tributyrin decreased MDA and H_2_O_2_ level, which indicates that tributyrin relieved the intestinal oxidative stress of weaned pigs.

Weaning often causes oxidative stress and then damages mitochondria [16]. As a result, damaged mitochondria release more ROS and then lead to ΔΨm collapse by the formation of permeability transition [44]. Therefore, higher mitochondrial ROS and lower ΔΨm are considered to be the markers of mitochondrial dysfunction [45, 46]. Although prior studies have found that butyrate can reduce intestinal oxidative stress, no evidence established the effect on intestinal mitochondrial function of pigs. In this experiment, we observed that tributyrin reduced mitochondrial ROS level and increased ∆Ψm of jejunum in comparison to the control. The findings suggested that tributyrin mitigated oxidative damage and enhanced mitochondria function. A previous study also found that, in high fat diet-induced obese mice, butyrate improved mitochondrial function reflected by the enhanced mitochondrial oxidative phosphorylation [47]. Li et al. [48] found butyrate inhibited the decrease of ∆Ψm in LPS-challenged cow mammary epithelial cell. Davis et al. [49] reported that butyrate reduced the mitochondrial ROS by increasing proton leak through upregulation of uncoupling protein 2. Russo et al. [19] demonstrated that in intestinal epithelial cells, butyrate was effective in controlling the increase of ROS levels in response to lipopolysaccharide.

The mtDNA was known to function significantly in oxidative phosphorylation and normal mitochondrial function [50]. The mtDNA content can reflect the mitochondrial function [50]. In this research, the mtDNA content of piglets fed tributyrin was higher than pigs in control, which was supported by Xing et al. [17], who reported that sodium butyrate restored the H_2_O_2_-induced decrease of mtDNA copy number in HepG2 cells. The result indicates that dietary tributyrin is quite helpful in attenuating mtDNA damage and improving mitochondrial function of weaned piglets. The alteration of mtDNA content is in accordance with the changes of multiple transcriptional key regulators involved in mitochondrial biogenesis [51]. *PGC-1α* is proven to be a major regulator of mitochondrial biogenesis [52]. *NRF-1* and *TFAM* are crucial for governing mtDNA replication and transcription during mitochondria biogenesis [53]. In the present study, the upregulated *NRF-1, PGC-1α* and *TFAM* mRNA abundances were observed in the jejunum of pigs fed tributyrin. Similarly, a previous study found that in oxidative injured HepG2 cells, sodium butyrate increased *PGC-1α* and *TFAM* mRNA levels [17]. Thus, tributyrin plays a relevant role in improving mitochondrial function of weaned pigs.

AMPK, a key energy sensor, functions in the regulation of cellular energy [54]. It has been shown that the activation of AMPK significantly modulates oxidative stress and mitochondrial function [55, 56]. Furthermore, AMPK activation inhibits mTOR, a serine/threonine protein kinase [21]. A large body of evidence suggest that the AMPK-mTOR pathway participated in the regulation of oxidative stress [22–24]. Wherefore, it is essential to investigate the effect of tributyrin on AMPK-mTOR signaling pathway. Our results revealed for the first time that tributyrin elevated the phosphorylation of AMPK and reduced phosphorylation of mTOR in intestine of pigs. These findings are consistent with previous results where SCFAs stimulate AMPK and inhibit mTOR in human colon cancer cells [57]. Similarly, Mollica et al. [58] found that in insulin-resistant obese mice, sodium butyrate increased liver AMPK activity, reduced ROS generation and improved mitochondrial function.

## Conclusions

To summarize, the current experiment proved that dietary supplementation with tributyrin enhances intestinal mucosa architecture, promotes the intestinal growth, extenuates oxidative stress and modulates the AMPK-mTOR signal pathway of weaned pigs.

## Data Availability

All data generated or analyzed during this study are available from the corresponding author on reasonable request.

## Abbreviations

AMPK: AMP-activated protein kinase
ASCT2: Na^+^ −dependent neutral amino acid transporter 2
BW: Body weight
CAT: Catalase
DCFH-DA: 2′,7′-dichlorohydro-fluorescein diacetate
GLUT2: Glucose transporter-2
GSH: Glutathione
GSH-Px: Glutathione peroxidase
H_2_O_2_: Hydrogen peroxide
MCT-1: Monocarboxylate transporter
MDA: Malondialdehyde
mt D-loop: Mitochondrial DNA-loop
mtDNA: Mitochondrial DNA
mTOR: Mammalian target of rapamycin
NRF-1: Nuclear respiratory factor-1
PepT1: Dipeptide transporter 1
PGC-1α: Peroxisomal proliferator-activated receptor-γ coactivator-1α
ROS: Reactive oxygen species
SCFA: Short chain fatty acid
SGLT1: Sodium glucose transport protein-1
SOD: Superoxide dismutase
TFAM: Mitochondrial transcription factor A
y^+^ LAT1: y^+^ L-type amino acid transporter 1
ΔΨm: mitochondrial membrane potential
